# Maternal Cancer Mortality and Orphanhood: A Neglected Global Health and Equity Challenge

**DOI:** 10.5334/aogh.5136

**Published:** 2026-02-17

**Authors:** Delfin Lovelina Francis

**Affiliations:** 1 Department of Public Health Dentistry, Saveetha Dental College and Hospitals, Saveetha University, SIMATS, Chennai, India

**Keywords:** maternal orphan, cancer mortality, breast cancer, cervical cancer, child outcomes, global health equity, LMICs, prevention, health systems, policy

## Abstract

*Background:* Maternal cancer mortality represents a growing but under-recognized global public health issue with profound consequences for surviving children. Breast cancer, cervical cancer, and other common malignancies disproportionately affect women in their reproductive years, leading to substantial psychosocial, health, and socioeconomic impacts for their children.

*Objective:* To synthesize current evidence on the global burden, determinants and consequences of maternal orphanhood due to cancer, and to identify prevention and policy opportunities aligned with existing health system goals and global cancer initiatives.

*Methods:* A structured literature search (2010–2025) was conducted across four databases using predefined keywords, with eligibility screening based on relevance to maternal cancer mortality and orphanhood outcomes. Evidence was analyzed under four thematic domains and interpreted comparatively using World Bank income classifications.

*Results:* An estimated 1.05 million children became orphans due to maternal cancer in 2020. The burden was greatest in Low and Middle Income Countries (LMICs) (83%), particularly in Asia and Africa (>80%), with the highest numbers in India, China, Nigeria, and Ethiopia. Breast, cervical, and upper gastrointestinal cancers are the leading causes. The majority of the affected children were ≥ 10 years old (69%). Maternal orphanhood was linked to poorer survival, mental health, education, and socioeconomic outcomes.

*Conclusions:* Maternal orphanhood from cancer highlights preventable inequities in women’s health, cancer control, and child support systems. Despite global initiatives, the burden remains largely unaddressed. Prioritizing equitable access to vaccination, screening, treatment, and social protection within national cancer policies is essential to reduce avoidable maternal deaths and protect affected children.

## Introduction

Globally, cancer is the second leading cause of death (approximately 10 million deaths/year), including 4.4 million among women in 2020 [1, 2]. The effects of cancer deaths have always received relatively less attention in the public health aspect, despite the fact that the direct health burden of cancer has been well-documented through metrics like death rates and disability-adjusted life years (DALY) [3]. This discrepancy is particularly significant in the case of maternal cancer mortality, which is not only an early cause of death but also has long-term effects on surviving children and families. Guida et al. recently estimated the scope of this problem, with an estimation of 1.05 million children becoming orphans due to maternal cancer death in 2020 [4]. According to these statistics, cancer accounts for 15% of all maternal orphanhood worldwide, making it a significant cause of this social and public health issue [5].

Maternal orphanhood needs high attention than the way it is merely treated as simple statistics. Maternal orphans are more likely to suffer from mental health issues, with higher rates of childhood mortality and high rates of infectious diseases and sexual violence, in addition to having less access to education [6–8]. By analyzing the cycles of poverty and health disparities across generations, these effects prevent the progress toward a number of sustainable development goals (SDGs), particularly SDG 3 (good health and well-being), SDG 4 (quality education), and SDG 10 (reduced inequalities) [9, 10].

## Methods

**Figure 1:**
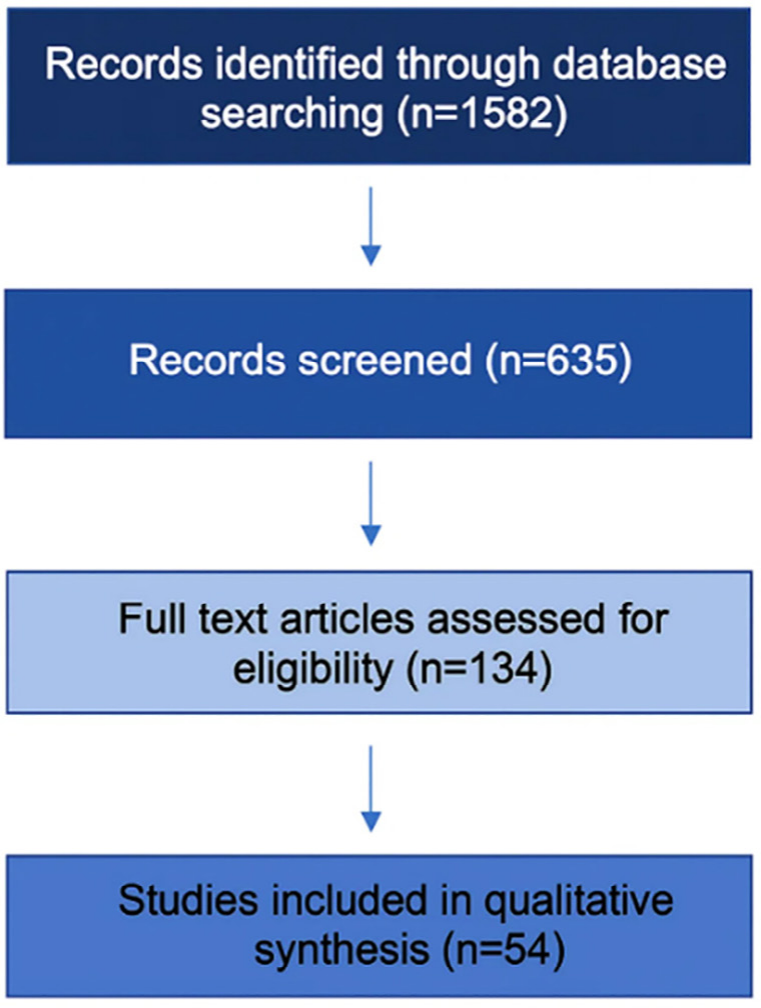
Methodology flowchart of the narrative review. The figure illustrates the structured approach used to identify, screen, and include studies in the qualitative synthesis (n = 54).

This narrative review utilized the SANRA (Scale for the Assessment of Narrative Review Articles) quality framework. A structured literature search was performed across PubMed, Scopus, Web of Science, and Google Scholar for articles published between January 2010 and February 2025, using combinations of the terms: maternal orphanhood, cancer mortality, breast cancer, cervical cancer, child health outcomes, psychosocial impact, Low and Middle Income Countries (LMICs), health inequities, and global burden of disease (Figure 1). Eligible sources included peer-reviewed original studies, systematic reviews, global health reports, and policy documents that examined maternal cancer mortality, the prevalence of orphanhood, associated health and social consequences for children, or prevention and policy strategies. Editorials, single case reports, commentaries, and studies unrelated to parental cancer or lacking orphan-related outcomes were excluded. Evidence was synthesized into four thematic domains: (1) global epidemiological burden and cancer-specific contributions to maternal orphanhood, (2) health, psychosocial, educational, and socioeconomic consequences for affected children, (3) inequities in prevention, diagnosis, and treatment across health systems, and (4) opportunities for prevention, mitigation, and policy integration aligned with global cancer control frameworks.

## Epidemiological Patterns of Maternal Cancer-Related Orphanhood

### Global distribution and regional variations

The distribution of maternal cancer orphans globally shows notable disparity. While Europe, America, and Oceania account for only 16% of the total, Asia alone accounts for nearly half and Africa for more than one-third of the count. It was also found that Ethiopia, Nigeria, Indonesia, China, India, and Pakistan account for two-fifths of the global total [11]. These geographic patterns reflect the in-depth relationships between fertility trends, cancer incidence and mortality rates, population demographics, and health system capacity. The percentage of women more affected than men by cancer at younger ages, when children are still minors, is very high in low-income countries, and this is mainly because of deaths from common cancers like cervix and breast cancer [12–14].

### Cancer-Specific contributions

Globally, breast, cervical, and upper gastrointestinal cancers together contribute to more than 50% of all maternal cancer orphans (Table 1). Breast and cervical cancer incidences are particularly significant from the perspective of public health prevention since screening, early detection, and treatment interventions can prevent both [15]. Regional variations in cancer-specific contributions reveal important epidemiological trends. In Eastern and Southern Africa, more mothers died from cervical cancer than from breast cancer, whereas in eastern Asia, the majority died from upper gastrointestinal cancer. In Europe, New Zealand, and Australia, respiratory cancers ranked second only to breast cancer [4]. These regional patterns reflect differences in risk factor prevalence, cancer etiology, and the capacity of the healthcare system.

**Table 1:** Maternal orphans by cancer type in 2020.

CANCER	ORPHANS	%	PREVENTABILITY
**Breast**	257,561	24.6	Moderate
**Cervical**	209,857	20.0	High
Upper GI	135,962	13.0	Low
Hematological	81,879	7.8	Low
Other female	63,054	6.0	Moderate
Lower GI	53,172	5.1	Moderate
Respiratory	50,580	4.8	High
Others	195,113	18.6	Variable
**Breast + cervix**	**467,418**	**44.6**	**WHO targets**

### Age patterns and fertility considerations

Most of the children orphaned were 10 years or older at the time of maternal death, and the rest were even younger. This age distribution has consequences for the timing of interventions and support needs because children at different developmental stages face different challenges following parental death [16]. Cancer deaths at maternal ages 35–49 accounted for the majority (63%) of maternal orphans, with a median at years 40–44. Peak reproductive ages, patterns of cancer incidence, and periods of child dependency are all intersected in this pattern. The number of orphans per 100 female cancer deaths varies dramatically by region, largely driven by fertility rates. While the ratio was less than 10 in low-fertility regions like Eastern Asia, Europe, and North America, it exceeded 110 orphans per 100 cancer deaths in Africa [4]. This highlights the influence of demographic contexts on the impact of maternal cancer mortality at the population level.

## Health and Social Consequences of Maternal Orphanhood

### Mortality and physical health impacts

Maternal loss has a major effect on a child’s survival. For instance, maternal orphans have higher childhood mortality rates than their peers in both high- and low-income settings. Data from a range of geographic locations support this effect: studies from Nordic countries show 1.25 times higher mortality rates among children whose parents died of cancer, while research from low-income countries shows a higher mortality at ages 2–10 [6, 7]. There are several factors that contribute to the higher mortality risk among orphans. Loss of maternal care affects nutrition, disease management, and access to medical care. Financial loss following a maternal death may force families to prioritize resources, which drives orphaned children to disadvantaged condition. Also, the child development and medical care-seeking behaviours are at stake due to financial instability in households and broken family bonds [17, 18].

### Mental health and psychosocial outcomes

Maternal orphans suffer a lifetime of psychological effects that they are more likely to suffer from mental health disorders, sexual violence, and suicide [19]. Longitudinal studies have shown that substance abuse, anxiety, depression, and PTSD were more common among those who lost a parent when they were young [20]. The prolonged illness and circumstances of cancer-related death may exacerbate psychological distress. Compared to sudden deaths, cancer deaths typically comprise a lengthy illness, a significant amount of treatment, and a gradual decline, during which time children may witness suffering and assume caregiving responsibilities. Due to the poor financial conditions and unsuccessful cancer treatment, both financial and emotional trauma are worsened, and the families may find it difficult to meet the needs of their children [21].

### Educational and economic consequences

According to research from sub-Saharan Africa, orphans traversing these situations are more likely to drop out of school early and get caught up in a poverty cycle, which results in poor school enrolment and completion rates than their peers who are not orphaned. These impacts persist into early adulthood and adolescence [22, 23]. The direct costs of education become unaffordable for orphans when household income is lost, children may be taken away to assist or take on household responsibilities, and psychological distress can impair cognitive function and academic performance [24]. These educational deficiencies result in fewer adult economic opportunities, perpetuating intergenerational poverty.

### Long-Term health trajectories

Orphanhood also has long-term impacts in the later life like, adolescent pregnancy, infectious and chronic diseases. The health impacts throughout life reflect both the behavioural pathways influenced by social circumstances and the direct biological effects of early adversity. Adolescent orphans are more likely to have multiple relationships, become pregnant, and have an early sexual debut, partly because of their financial vulnerability and lack of parental supervision. These result in higher rates of HIV and other STDs, particularly in high-prevalence settings. Chronic stress brought on by orphanhood may also affect physiological systems for a long time, raising the risk of metabolic and cardiovascular diseases in later life [25].

## Inequities in Maternal Cancer-Related Orphanhood

### Socioeconomic gradients

**Figure 2:**
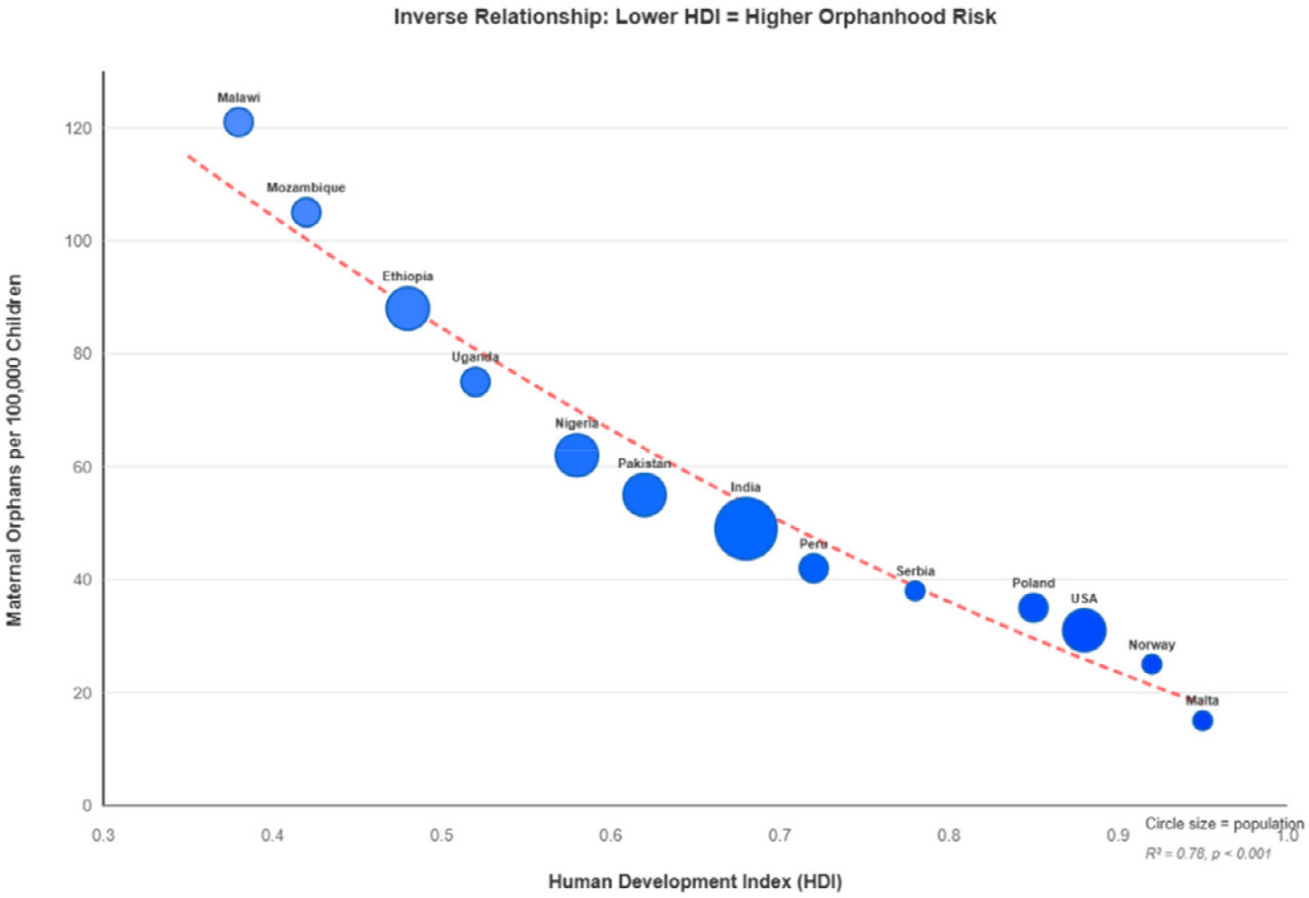
Maternal cancer orphanhood risk by human development index.

Maternal cancer orphanhood exhibits pronounced socioeconomic gradients operating at individual, national, and global levels. Countries with lower Human Development Index (HDI) had greater numbers of newly orphaned children, ranging from 15 in Malta to 121 in Malawi per 100,000 children. Multiple intertwined factors in the disparities in cancer prevention, detection, treatment, and survival are reflected in this pattern (Figure 2). Despite lower incidence, countries that have a lower HDI have higher cancer mortality rates among adolescents, which reflects limited access to early detection and treatment. This negative correlation between orphanhood risk and development highlights how cancer mortality exacerbates already-existing global health disparities [4, 13].

Studies from Africa showed that women with poor education are diagnosed with untreatable breast cancer with low survival rates compared to women with higher educational levels. Also, these women have more children who eventually become maternal orphans and get caught in the vicious cycle [26, 27]. This pattern creates an intergenerational cycle wherein disadvantaged women face higher mortality risk while their children who are already at higher vulnerability lose maternal support and protection.

### Geographic and healthcare access disparities

In most African countries, the cervical cancer mortality rates at reproductive ages were 2–8 times higher than the global average, combined with high fertility rates. However, few low-income countries demonstrated a different pattern, while fertility was not exceptionally high, breast cancer mortality rates were 3–4 times higher than the global average between 15 and 54 years of age [4]. These differences highlight how orphanhood risk is influenced by various combinations of cancer patterns, healthcare access, and demographic characteristics. However, the high cancer mortality rate in LMICs is largely caused by deficient healthcare infrastructure. Curative treatment is hampered by a lack of surgical capability, radiotherapy facilities, diagnostic pathology, and systemic treatment [28, 29]. Access barriers are particularly severe for economically disadvantaged women due to geographic distance, transportation costs, and opportunity costs of seeking care, even in cases where services are available [30].

## Prevention Opportunities: Focus on Cervical Cancer and HPV Vaccination

### The preventable burden of cervical cancer

Scientific data show that 210,000 children were orphaned in 2020 due to cervical cancer worldwide [4]. This prominence is especially noteworthy because, when detected early, cervical cancer is one of the most treatable and preventable cancers. About 90% of cervical cancer deaths take place in LMICs, where access to treatment is restricted and screening rates are still low [31]. Despite lower incidence rates, age-standardized mortality rates in Eastern and Southern Africa are significantly higher than in high-income nations, indicating serious shortcomings in early detection and treatment [32].

From an orphanhood prevention perspective, cervical cancer represents a critical target. Unlike many cancers with complex multifactorial etiology, cervical cancer has a necessary cause with persistent infection due to high-risk human papillomavirus (HPV) types, against which highly effective vaccines exist [33].

### HPV vaccination as primary prevention

HPV vaccination represents the most effective primary prevention method against cervical cancer. When given prior to sexual debut, three WHO-prequalified vaccines (bivalent, quadrivalent, and nonvalent) show over 90% efficacy against vaccine-type HPV infections and high-grade cervical intraepithelial neoplasia. Cervical abnormalities, HPV infection, and cervical cancer incidence are all on the decline, according to real-world data from nations with high vaccination rates [34]. Vaccinating young girls against HPV before they start having sex will prevent cancer in future generations of women and, consequently, the orphanhood of their offspring. According to modeling studies, the WHO Cervical Cancer Elimination Initiative aims to vaccinate 90% of girls by the age of 15, which could avert millions of cervical cancer cases and deaths in the ensuing decades [35].

Single-dose HPV vaccination schedules have revolutionary implications for global implementation, according to recent evidence. Single-dose vaccination offers long-lasting protection equivalent to multi-dose schedules, making delivery easier and increasing coverage achievability, as shown by the IARC India study and other trials [36]. WHO updated its recommendations in 2022 in response to this evidence, supporting single- or two-dose schedules based on immunocompetence status and age [37]. HPV vaccine coverage is still below ideal worldwide, despite its proven efficacy and safety. Only 15% of eligible girls globally had received the full HPV vaccination as of 2020, with significant differences between high-income and LMIC nations [38].

### Secondary prevention through screening

Cervical precancer screening and treatment are still crucial for today’s adult women who are past the vaccination age. Cervical cancer mortality rates can be significantly decreased in adult women by screening for and treating cervical intraepithelial neoplasia. Conventional cytology-based screening, however, necessitates a large infrastructure, knowledgeable staff, and follow-up systems that are frequently unavailable in environments with limited resources. Opportunities to increase coverage in LMICs are presented by recent developments in screening technology. Single-visit screen-and-treat strategies are made possible by HPV DNA testing as primary screening, which also exhibits superior sensitivity to cytology and can be carried out on self-collected samples [39]. Visual inspection with acetic acid (VIA), though less sensitive than HPV testing, offers a low-cost option suitable for low-resource settings when coupled with immediate treatment.

In addition to 90% treatment of precancerous lesions and 90% management of invasive cancer, the WHO Cervical Cancer Elimination Initiative aims to achieve 70% screening coverage by age 35 using a high-performance test. It would be necessary to significantly expand screening programs in order to meet these goals, especially in high-burden LMICs where coverage is frequently below 20% [40].

## Breast Cancer: Complex Challenges and Intervention Opportunities

### Breast cancer as leading cause of maternal orphans

In 2020, breast cancer accounted for 258,000 orphaned children worldwide, making it the leading cause of new maternal orphans (25%). This is in line with the fact that breast cancer is the most common cancer diagnosed and the primary cause of cancer-related deaths among women globally. Unlike cervical cancer, breast cancer incidence continues rising globally, particularly in transitioning countries experiencing fertility decline, dietary changes, and reduced physical activity. Compared to cervical cancer, the situation of preventing breast cancer fatalities is more complicated. As fertility and lifestyle changes continue, incidence rates at postmenopausal ages are increasing, and any further fatalities would result in elder mother orphans. Currently, no interventions demonstrate comparable effectiveness to HPV vaccination for primary breast cancer prevention in the general population [41].

### Improving breast cancer survival in LMICs

Improving breast cancer survival in LMICs becomes the need of the hour to attain the maximum levels of the high-income nations to reduce maternal orphans linked to breast cancer. Due to late diagnosis and limited access to treatment, five-year survival rates are below 50% in many LMICs but surpass 85–90% in high-income countries. Cancer diagnostics, surgical, chemotherapy, and radiation therapy facilities, and professional training have to be improved significantly to improve early detection and prompt, high-quality treatment.

The WHO Global Breast Cancer Initiative provides a framework for enhancing breast cancer management and focuses on the three important aspects of health promotion for early detection, timely diagnosis, and comprehensive breast cancer management [20, 21]. In order to improve the outcomes in LMICs, it is important to implement community awareness programs that encourage early detection, train primary healthcare providers in clinical breast examination and referral protocols, establish diagnostic services with immunohistochemistry to ensure surgical capacity for mastectomy and breast-conserving surgery, and provide systemic therapies such as hormonal therapy, chemotherapy, and targeted agents when necessary [40].

### Financial toxicity and orphanhood risk

The significant costs associated with cancer treatment lead to poverty in the family. As a result, the orphans struggle to complete their education, are forced to live in poverty, and are more likely to experience serious health problems throughout their lives. This leads to the enhancement of poverty cycle and disadvantage to generations.

All the poor outcomes are both a result of and a cause of the financial toxicity of cancer care. The household’s savings are depleted by lost income, transportation, lodging, and direct medical expenses, along with forced asset sales or debt accumulation. These economic shocks continue after death, leaving orphaned children in households with fewer resources and less money to spend on health and education. In order to end this cycle, it is crucial for financial protection systems and universal health coverage. Improved treatment completion, survival, and decreased financial burden have been shown in nations that have implemented cancer-focused health financing programs [42].

## Policy Implications and Support Strategies

### WHO global initiatives: Implementation imperatives

WHO Global Breast Cancer Initiative and Cervical Cancer Elimination Initiative aim to invest in and speed up the expansion of the number of maternal orphans caused by breast and cervical cancers. For national cancer control programs, these initiatives set evidence-based frameworks, goals, and metrics. The 90–70–90 targets (90% HPV vaccine coverage, 70% screening coverage, and 90% precancer and cancer treatment) offer precise standards for the eradication of cervical cancer. According to modeling, reaching these goals on a global scale could prevent hundreds of thousands of maternal orphans and significant cervical cancer deaths in the coming decades. Political commitment, multi-sectoral coordination, consistent funding, and accountability systems are necessary for country-level implementation (Table 2).

**Table 2:** Key interventions summary.

INTERVENTION	EVIDENCE	EFFICACY	FEASIBILITY	PROJECTED IMPACT
**HPV vaccination**	High	90%–100% vs HPV 16/18	Moderate	160 K orphans prevented/year by 2050
**HPV screening**	High	60%–80% reduction	Moderate	100 K orphans prevented/ year
**VIA + cryotherapy**	Moderate	25%–35% reduction	High	50 K orphans prevented/ year
**Breast early detection**	Moderate	20%–30% mortality reduction	Moderate-Low	50–70 K orphans prevented/ year
**Breast comprehensive treatment**	High	85%–90% 5-yr survival	Low	130 K orphans prevented/ year
**UHC + financial protection**	Moderate	Improves treatment completion	Context-dependent	Enables all interventions
**Cash transfers (orphans)**	High	Improves education, health	Moderate	Mitigates orphanhood impact
**Psychosocial support**	Moderate	Reduces mental health burden	High	Long-term well-being

**Evidence levels:** High = RCTs/systematic reviews; Moderate = observational/cohort; Low = limited data.

**Feasibility:** Based on resource requirements, infrastructure, and implementation complexity.

In LMICs, where late-stage presentation is more common, the Global Breast Cancer Initiative highlights the value of early diagnosis and comprehensive management in enhancing survival. Context-appropriate implementation pathways must take into account the various capacities of the healthcare system, resource limitations, and sociocultural contexts [43].

### Orphan support programs: Lessons and applications

Lessons from HIV orphan support programs are relevant, even though HIV orphans and maternal orphans from cancer are very different, as orphans resulting from HIV are typically double orphans, significantly younger, and concentrated in sub-Saharan Africa. In the early phases of the epidemic, a large number of these orphans had HIV. Recent research showed that rather than receiving care in an institution, the majority of orphans can and should be supported within their families and communities.

Cash transfer programs have well improved school enrolment, nutritional status, and health outcomes, providing orphan care households with consistent financial support. Child development and mental health are improved by parenting interventions that increase caregiver capacity, and resilience is enhanced by psychosocial support that addresses trauma, grief, and stigma. Most programs serve children orphaned by any cause, including cancer orphans, in order to prevent stigmatization. This all-inclusive strategy acknowledges shared vulnerabilities and permits focused interventions when necessary. In order to address educational barriers, some cancer-specific foundations, like the Lalla Salma Foundation in Morocco, offer scholarship programs for cancer orphans [44].

### Integrating orphanhood prevention into cancer control

Orphanhood prevention should be specifically included in cancer control strategies as a driving force behind action and a quantifiable result. Impact evaluations of cancer interventions should measure not only the number of deaths avoided but also the number of orphans. The perceived value of treatments aimed at cancers affecting women of reproductive age is increased by this framing.

Data on parental status at death should be gathered by cancer registries and vital statistics systems so that trends in maternal orphans can be tracked and the effects of interventions can be assessed. Such information would help allocate resources, highlight high-burden groups that need more assistance, and monitor the achievement of orphanhood reduction goals. Family-centered care can be incorporated into clinical oncology practice to meet the needs of patients’ children. Cancer care for parents of young children should include routine referrals to social services, family communication counseling, and pediatric psychological support. Financial provisions for children and guardianship arrangements should be covered in advance care planning [45].

### Strengthening health systems and addressing social determinants

Sustainable reductions in maternal cancer orphanhood require a complete strengthening of the health system that includes cancer prevention, early detection, diagnosis, treatment, and palliative care throughout the care continuum. Essential cancer services must be integrated into universal healthcare packages so that all demographic groups can afford them, and addressing the social determinants of health that lead to cancer disparities is equally crucial. Education for women typically lowers cancer risk behaviors with an increase in healthcare utilization, facilitates economic participation, and enhances health literacy. At the same time, social protection systems, gender parity, and poverty reduction decrease vulnerability and enhance population health outcomes [46].

### Conceptual framework and intervention opportunities

**Figure 3:**
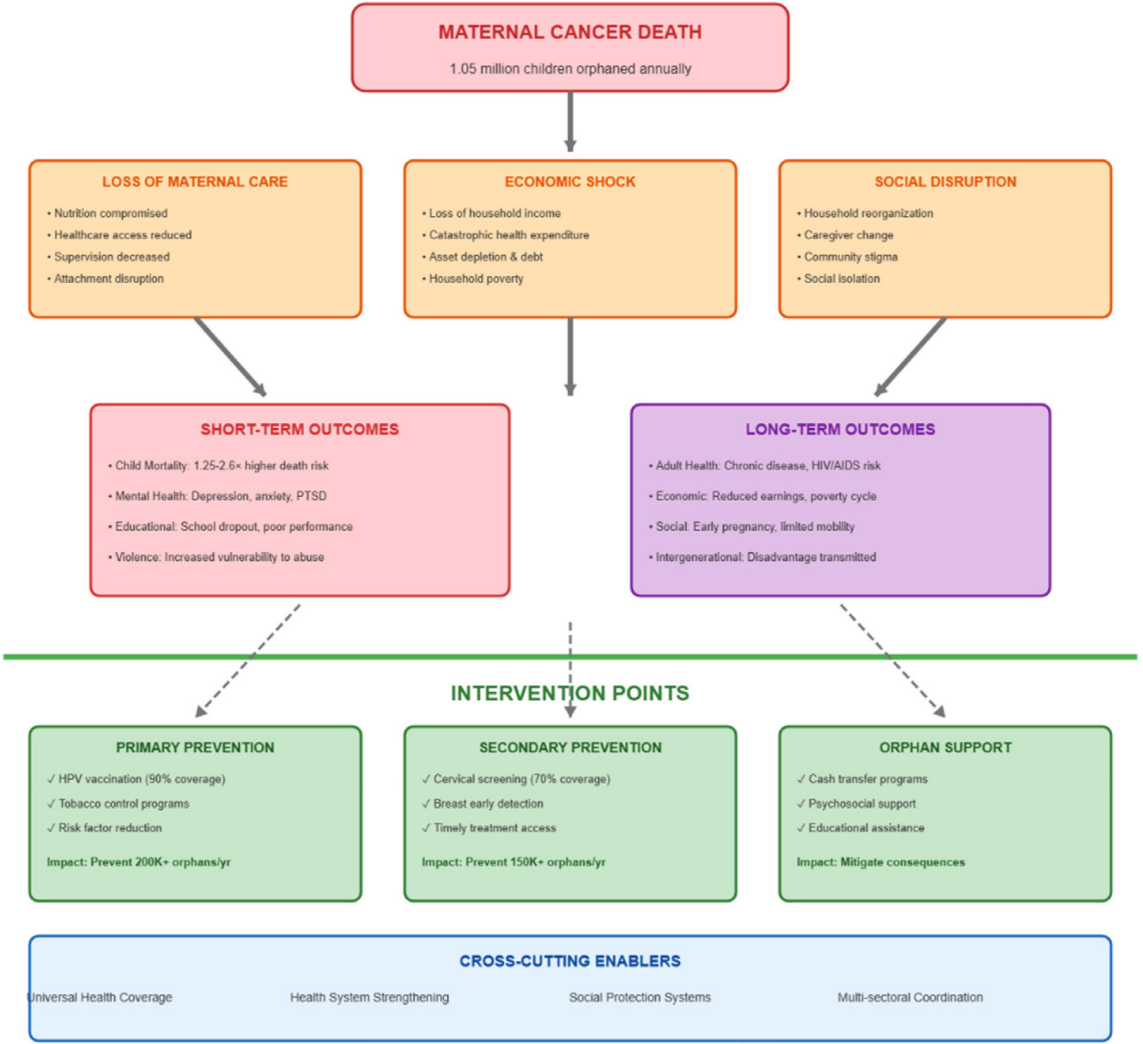
Conceptual framework: pathways from maternal cancer death to child outcomes and intervention points.

To understand the complex pathways from maternal cancer mortality to child outcomes and identify strategic intervention points, we developed a conceptual framework (Figure 3). This framework demonstrates how the death of a mother from cancer causes immediate repercussions in three areas: social disruption, economic shock, and loss of maternal care. These repercussions then cascade into both short-term and long-term child outcomes. Crucially, the framework distinguishes three levels of intervention: tertiary support (reducing the impact on orphans), secondary prevention (early detection and treatment), and primary prevention (preventing cancer deaths). Implementation at all intervention levels is facilitated by cross-cutting enablers such as social protection, health system strengthening, universal health coverage, and multi-sectoral coordination (Figure 3).

### Research gaps and future directions

Despite growing recognition of maternal cancer, there are still significant research gaps. The modeling methods used to generate the current estimates must be verified by prospective cohort studies that follow cancer-orphaned children in different settings. These studies should assess health trajectories, economic conditions, educational outcomes, and protective factors that promote resilience. Intervention research is needed to develop and evaluate support programs for cancer orphans and their families. Practical trials comparing different service delivery models, benefit packages, and targeting strategies would inform program design and scale-up.

Health economics research that measures the entire societal costs of maternal cancer deaths, such as lost productivity, the transmission of poverty across generations, and the support needs for orphans, would bolster the investment case for cancer prevention and treatment. Cost-effectiveness analyses of cancer interventions should incorporate the benefits of orphanhood prevention that are currently excluded from most evaluations. Research on overcoming vaccine hesitancy, increasing screening uptake, and ensuring treatment completion would increase the effectiveness of the program. Since cancer-related paternal orphanhood is still largely unmeasured, a thorough evaluation necessitates expanding techniques for estimating paternal orphans and analyzing the differences in the effects of maternal versus paternal loss [47, 48].

## Conclusion

Maternal cancer-related orphanhood is a neglected global health and development challenge, with over one million children losing their mothers to cancer every year. Breast and cervical cancers, which are both preventable, detectable early, and treatable effectively, account for the majority of these deaths. Supported by national plans that expand HPV vaccination, cervical screening, and equitable access to high-quality cancer treatment, the WHO Global Breast Cancer Initiative and Cervical Cancer Elimination Initiative must be fully implemented as a top priority for policy action. Recent studies highlight the need to reduce preventable maternal cancer deaths while also preserving the rights, health, and socioeconomic well-being of orphaned children. For affected families, governments and international partners should provide cross-sectoral support networks, ensure financial protection, and incorporate orphanhood prevention into cancer control frameworks. Supporting these programs is in line with the SDGs and demonstrates a moral and policy commitment to global health equity and intergenerational justice.

## Verification of Authors

It is verified that all authors had access to the data and a role in writing the manuscript.
